# DNA Nicks Promote Efficient and Safe Targeted Gene Correction

**DOI:** 10.1371/journal.pone.0023981

**Published:** 2011-09-01

**Authors:** Luther Davis, Nancy Maizels

**Affiliations:** 1 Department of Immunology, University of Washington School of Medicine, Seattle, Washington, United States of America; 2 Department of Biochemistry, University of Washington School of Medicine, Seattle, Washington, United States of America; 3 Northwest Genome Engineering Consortium, Seattle, Washington, United States of America; National Cancer Institute, United States of America

## Abstract

Targeted gene correction employs a site-specific DNA lesion to promote homologous recombination that eliminates mutation in a disease gene of interest. The double-strand break typically used to initiate correction can also result in genomic instability if deleterious repair occurs rather than gene correction, possibly compromising the safety of targeted gene correction. Here we show that single-strand breaks (nicks) and double-strand breaks both promote efficient gene correction. However, breaks promote high levels of inadvertent but heritable genomic alterations both locally and elsewhere in the genome, while nicks are accompanied by essentially no collateral local mutagenesis, and thus provide a safer approach to gene correction. Defining efficacy as the ratio of gene correction to local deletion, nicks initiate gene correction with 70-fold greater efficacy than do double-strand breaks (29.0±6.0% and 0.42±0.03%, respectively). Thus nicks initiate efficient gene correction, with limited local mutagenesis. These results have clear therapeutic implications, and should inform future design of meganucleases for targeted gene correction.

## Introduction

Targeted gene correction (TGC) is a powerful approach to gene therapy of monogenic disorders [1], [2]. TGC corrects a mutation by transiently providing a correct DNA template for repair by homologous recombination. The efficiency of TGC is naturally low, but can be greatly improved by creating a DNA break near the mutation to be corrected [3]. Breaks are generated by meganucleases, which recognize long target sites (16–24 bp) with good but imperfect sequence-specificity [4], [5]. Three classes of meganucleases are in current use: homing endonucleases, which promote intron mobility in microorganisms and naturally recognize long motifs; and zinc-finger and TALE nucleases, built by linking modular sequence-recognition domains to the FokI nuclease domain [6]–[8].

TGC has clear advantages relative to traditional gene therapy, which provides a functional gene copy as a transgene. TGC preserves regulatory sequences necessary for proper gene expression, and eliminates the danger of insertional activation of proto-oncogenes that can accompany integration of a therapeutic transgene carried on a viral vector [9]. However, initiation of TGC with a double-strand break (DSB) has a potential hazard: if repair occurs by nonhomologous end-joining (NHEJ) pathways, rather than homologous recombination, TGC may be accompanied by mutations or translocations at the target site and elsewhere in the genome. In addition, as these meganucleases do not cleave with absolute sequence-specificity, off-target cleavage may occur at sites other than the disease gene [5]. Off-target cleavage poses an especially significant risk as these events may be difficult to identify.

We have postulated that DNA nicks might provide a way to initiate TGC without the inherent risk of genomic instability that is associated with DSBs. We previously showed that a DNA “nickase” derived from the I-AniI LAGLIDADG homing endonuclease recognized the same 20 bp target sequence as the parental “cleavase”, and initiated correction of a chromosomal mutation [10]. Here, we compare efficiency of TGC initiated by nicks and DSBs at the same target site, concurrently assaying local mutagenesis to provide a measure of the safety of each approach. We show that nicks promote TGC with few accompanying deleterious events, while DSBs cause significant levels of heritable genomic alterations both locally and elsewhere in the genome. Measuring efficacy as the ratio of TGC to local frameshift mutations, nick-initiated TGC exhibited 70-fold greater efficacy than did DSB-initiated TGC. Thus, DNA nicks are proficient at promoting TGC and essentially immune to deleterious repair. These results have clear therapeutic implications, as they should inform design and engineering of all three classes of meganucleases currently being adapted for targeted gene correction.

## Results

### Nicks promote TGC with very few local NHEJ events

In order to compare the frequency of local NHEJ inadvertently caused by initiation of TGC by nicks or DSBs, we used the I-AniI nickase and cleavase in conjunction with a dual reporter of TGC and NHEJ. The nickase was derived from the I-AniI homing endonuclease by substitution of a methionine for a lysine (K227M) in one of the two active sites. The K227M substitution inactivates one active site and results in an enzyme with strong nicking activity but no detectable DSB formation [10].

**Figure 1:**
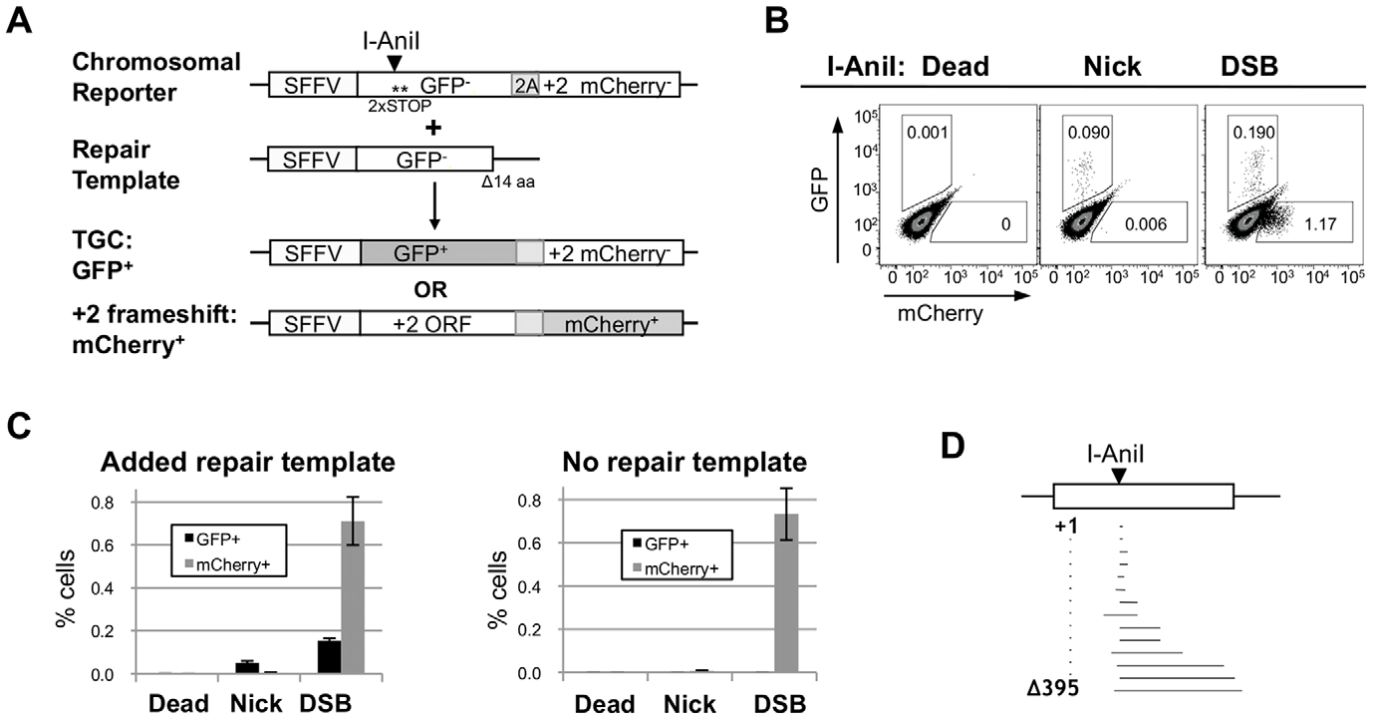
Nicks promote TGC with little accompanying local mutagenesis. (A) I-AniI Traffic Light reporter assay for TGC. The chromosomal reporter consists of an SFFV promoter driven defective GFP gene, containing an I-AniI recognition sequence (arrowhead) and two stop codons near the N-terminal (asterisks), linked by a T2A sequence to a mCherry gene in the +2 reading frame. The exogenous repair template is a defective GFP gene with an intact N-terminal but lacking 14 residues at the C-terminal. TGC results in GFP^+^ expression, and +2 frame shifts at the I-AniI site result in mCherry+ expression. (B) Results of a representative experiment assaying TGC (GFP^+^) and +2 frame shifts (mCherry^+^) caused by expression of mutant I-AniI, I-AniI nickase or I-Ani cleavase in the presence of a repair template. (C) Comparison of TGC (GFP^+^) and +2 frame shifts (mCherry^+^) promoted by expression of catalytically inactive I-AniI (Dead), I-AniI nickase (Nick) or I-Ani cleavase (DSB), in the presence (left) or absence (right) of a repair template. Mean and standard error of the mean (SEM) of three to six (left) and eight (right) independent experiments are shown. (D) Diagram of the length and orientation of 16 independent +2 frameshift mutations at I-AniI site leading to mCherry+ expression (sequences alignment is in Fig. S1).

The Traffic Light reporter [11] is designed to detect both TGC and NHEJ (+2 frame-shift) at a single meganuclease recognition sequence (Fig. 1A). The reporter carries a single translation start site and a defective GFP coding sequence bearing two in-frame stop codons, one within and the second immediately adjacent to an I-AniI cleavage site. A mCherry gene is fused to GFP in the +2 reading frame by a T2A translational linker. The +2 reading frame of GFP contains no stop codons, so if deletion or insertion causes a +2 frame shift, mCherry will be translated to generate mCherry^+^ cells. Thus GFP^+^ cells report on gene correction and mCherry^+^ cells report on local frameshift mutations (an underestimated since only one of three reading frames is detected).

Stable 293T cell transductants bearing the Traffic Light reporter were transiently transfected with constructs expressing I-AniI meganuclease in the presence or absence of a repair template, and levels of GFP^+^ and mCherry^+^ cells quantitated 3 days posttransfection by flow cytometry. As shown by one representative experiment, nick-initiated TGC produced GFP^+^ cells but few mCherry^+^ cells; while DSB-initiated TGC produced GFP^+^ cells and many mCherry^+^ cells (Fig. 1B). Compilation of data from eight independent transfections (Fig. 1C, left) showed that essentially no GFP^+^ or mCherry^+^ cells were present following expression of inactive I-AniI (0.001±0.000%, 0.001±0.000%, respectively). Targeted nicks or DSBs promoted significant frequencies of GFP^+^ cells (0.051±0.008% and 0.154±0.011%, respectively), but nicks produced 140-fold fewer mCherry^+^ cells than did DSBs (0.005±0.001% and 0.711±0.112%, respectively; p = 4.0×10^−4^, two-tailed t-test).

GFP^+^ cells were the products of TGC, as they were not evident following expression of a meganuclease in the absence of a repair template (0.001±0.000%, inactive I-AniI; 0.000±0.000%, nick; 0.001±0.000%, DSB; Fig. 1C, right). Frequencies of mCherry^+^ cells were unaffected by the absence of a repair template (0.000±0.000%, in active I-AniI; 0.007±0.002% nick; 0.733±0.120%, DSB). Sequencing verified that 16 events leading to mCherry expression were the expected +2 frameshifts formed by NHEJ and included one insertion (1 bp) at the target site, 11 deletions (2–353 bp), and four complex deletion/insertions (Figs. 1D and S1). Thus, DSBs but not nicks cause local deletions.

We conclude that nicks initiate TGC nearly as efficiently as DSBs but with many fewer accompanying local NHEJ events.

### The frequency of TGC reflects meganuclease expression levels

**Figure 2:**
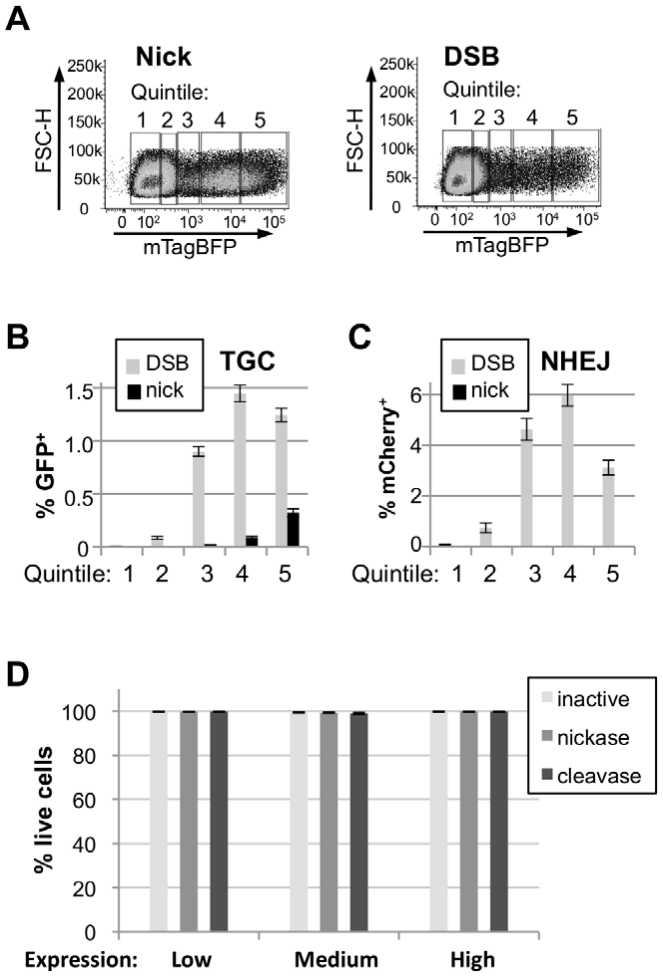
Frequency of TGC and NHEJ as a function of I-AniI expression levels. (A) Representative experiment showing the five fluorescent gates (left) applied to cells transfected with I-AniI nickase-T2A-mTagBFP (left) or I-AniI cleavase-T2A-mTagBFP (right). (B) Comparison of frequencies of TGC (GFP^+^) initiated by DNA nicks or DSBs in each quintile of meganuclease expression. The mean and SEM of eight independent transfections are graphed. (C) Comparison of frequencies of NHEJ (mCherry^+^) initiated by the I-AniI nickase or cleavase in each quintile of meganuclease expression. Mean and SEM of eight independent transfections are shown. (D) Comparison of cell viability three days post-transfection with I-AniI dead, nickase or cleavase expression plasmids. Cells were gated into three expression bins (Low, Medium, High) based on mTagBFP fluorescence and viability was assayed. The Mean and SEM of three independent transfections are shown.

To ask if the frequency of TGC and NHEJ varies with meganuclease expression levels, we transfected cells bearing the Traffic Light reporter with constructs co-expressing I-AniI (inactive, nickase or cleavase) and mTagBFP [12] from the same promoter and separated by a T2A translational linker to achieve approximately stoichiometric expression [13]. Transfectants were gated by flow cytometry into five bins (quintiles) according to mTagBFP fluorescence (Fig. 2A) and the frequency of GFP^+^ and mCherry^+^ cells in each bin determined. The results of a series of independent experiments showed that the frequency of TGC increased with increasing levels of I-AniI nickase expression, with the highest frequency (0.322±0.037%) occurring in cells expressing the highest levels of enzyme (Fig. 2B and Table S2). In contrast, the frequency of TGC was highest at the fourth quintile of I-AniI cleavase expression (1.45±0.08%), and decreased in the fifth quintile (Fig. 2B and Table S2). In each case, TGC frequencies in the optimal quintile were significantly greater than in the total population (nickase, 6-fold; cleavase, 9-fold). We conclude that frequencies of nick-initiated TGC increased with increased enzyme expression while frequencies of DSB-initiated TGC frequencies peaked at less than maximal levels of enzyme expression.

Comparison of the frequency of mCherry^+^ cells (NHEJ) for each quintile of enzyme expression showed that I-AniI nickase expression was accompanied by low frequencies of NHEJ, which varied little with enzyme expression levels (Fig. 2C). In contrast, even the lowest level of I-AniI cleavase expression (first quintile) was accompanied by a significant frequency of NHEJ, exceeding frequencies observed at any level of nickase expression. Cleavase-initiated NHEJ increased with increasing enzyme levels, peaking in the fourth quintile (Fig. 2C and Tables S1 and S2). Thus, in cells expressing I-AniI cleavase, the NHEJ and TGC frequencies co-varied (Fig. 2B and C) and decreased at the highest expression levels, suggesting that high enzyme levels may inhibit productive cleavage. Notably, nick-initiated TGC occurred at only 4-fold lower frequency than DSB-initiated TGC, and with very low levels of associated NHEJ.

The reduced level of cleavase-induced NHEJ and TGC at the highest expression levels led us to ask whether viability was impacted by cleavase expression. Viability was measured 3 days posttransfection using an assay of membrane integrity, and shown to be >98.5% even at the highest expression levels (Fig. 2D). We conclude that none of the I-AniI derivatives used here had a deleterious effect on cell viability.

### Nicks initiate gene correction with much higher efficacy than do DSBs

**Figure 3:**
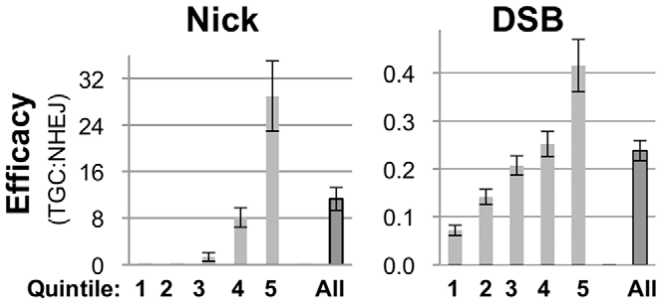
Nicks initiate TGC with much higher efficacy than do DSBs. Efficacy, measured as the ratio of TGC (GFP^+^) to NHEJ (mCherry^+^), in each quintile of expression of I-AniI nickase-T2A-mTagBFP or I-AniI cleavase-T2A-mTagBFP. The mean and SEM of eight independent transfections are shown.

We defined efficacy of nick- or DSB-initiated gene correction as the ratio of TGC/NHEJ — i.e. the ratio of GFP^+^/mCherry^+^ cells. Maximum efficacy for both nicks and DSBs was reached at the highest levels of enzyme expression (Fig. 3, note the y-axis scales differ by a factor of 70). Strikingly, nicks initiated TGC with 70-fold greater efficacy than DSBs at the highest levels of meganuclease expression (29.0±6.0% and 0.42±0.03%, respectively; p = 0.003) and 50-fold greater efficacy in the total populations (12.0±1.8% compared to 0.24±0.02%, p = 0.0004; Fig. 3 and Table S2).

### Nick-initiated TGC is associated with few second-site events

**Figure 4:**
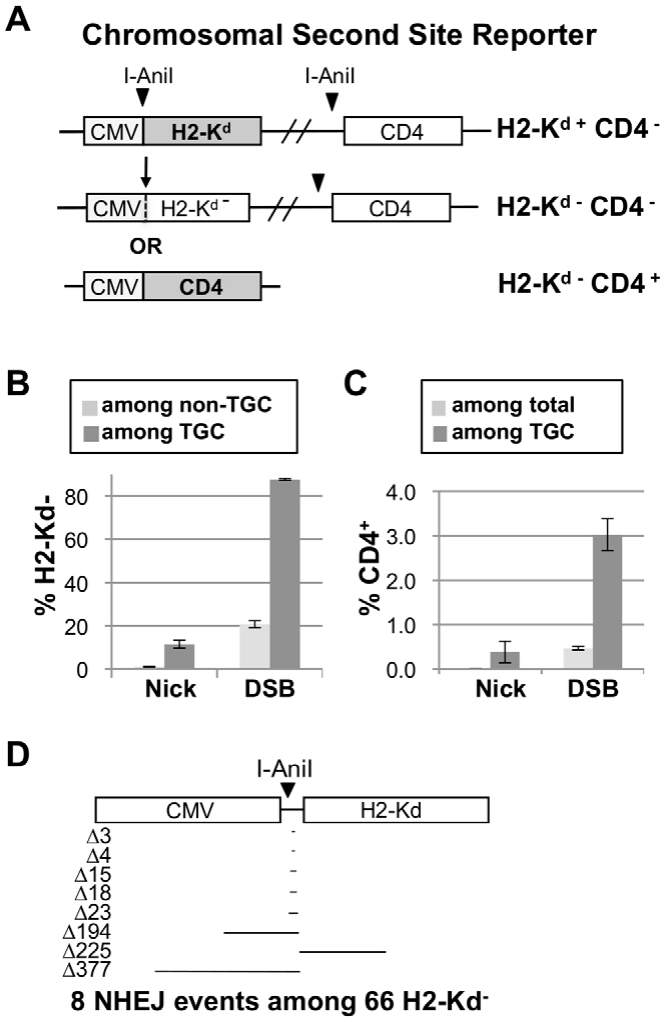
Nick-initiated TGC is associated with few second-site events. (A) Chromosomal second-site reporter, carrying a CMV promoter (CMV), I-AniI site, a gene encoding the H-2K^d^ cell surface protein, and a gene encoding the CD4 cell surface protein downstream of a second I-AniI site (top) Cells expressing this reporter are H-2K^d+^ CD4^−^. Cleavage at the upstream I-AniI site followed by NHEJ-mediated sequence loss can result in loss of H-2K^d^ expression (middle). Cleavage at both I-AniI sites can result in deletion of the region between these two sites that carries the H-2K^d^ and expression of CD4^+^ (bottom). (B) Comparison of frequencies of loss of H2-K^d^ expression among cells that had or had not undergone TGC initiated by nicks or DSBs. Mean and SEM of six independent transfections are shown. (C) Comparison of frequencies of gain of CD4^+^ expression among cells that had and had not undergone TGC initiated by nicks or DSBs. Mean and SEM of four independent transfections are shown. (D) Diagram of the length and orientation of the 8 deletions detected among 66 sequences from a sorted population of H2-K^d^-negative cells. Only 3 of the deletions included either coding or promoter sequence.

To determine whether the advantage in efficacy of nick-initiated TGC extended beyond the target site, we constructed cell lines carrying two stably integrated chromosomal reporters to measure TGC and second-site events in the same cells. These dual reporters can measure both on-target TGC and off-target events in a single cell, while TGC and NHEJ are mutually exclusive events at the Traffic Light reporter. In the dual reporter system, the TGC reporter was a GFP gene carrying two in-frame STOP codons just downstream of the I-AniI site near the 5′ end of the coding sequence (Fig. S2). The second site reporter, derived from an NHEJ reporter developed in the Lopez laboratory [14], carries one I-AniI site upstream of the gene for the H2-K^d^ cell surface protein, and another just downstream of a gene encoding the CD4 cell surface protein. NHEJ-mediated deletions following cleavage at the upstream I-AniI site can result in loss of H2-K^d^ expression; and NHEJ-mediated loss of the sequence between the two I-AniI sites will result in expression of CD4^+^ accompanied by loss of H2-K^d^ expression (Fig. 4A).

Frequencies of nick-initiated or DSB-initiated TGC were 0.12±0.01% and 0.83±0.12% respectively in cells carrying both reporters (Table S3). The frequencies of H2-K^d^ loss associated with targeted nicks and DSBs were 20-fold different among cells in which TGC had not occurred (GFP^−^; 1.05±0.21% and 20.77±1.56%, respectively, p = 4.55×10^−5^); and 8-fold different among cells in which TGC had occurred (GFP^+^; 11.51±1.81% and 87.73±0.46%, p = 3.44×10^−8^, Fig. 4B). The frequencies of tandem events leading to CD4^+^ expression associated with nicks or DSBs were similarly very different in total cell populations (−0.01±0.02% and 0.47±0.04%, respectively; p = 0.0001) and 8-fold different among cells in which TGC had occurred (0.39±0.24% and 3.03±0.36% respectively; p = 0.001, Fig. 4C). Thus, the efficacy advantage of nick-initiated TGC extends to sites other than the target of gene correction.

### DSBs result in loss of H2-K^d^ expression not associated with sequence loss

To determine if deletions within the promoter or coding sequence accounted for loss of H2-K^d^ expression, we sequenced the region surrounding the I-AniI cut site between the promoter and H2-K^d^ coding sequence from a sorted population of cleavase-induced H2-K^d^-negative cells. Of 66 sequences examined, only eight carried deletions and only three of these were sufficiently long to affect H2-K^d^ expression (Fig. 4D). Thus, most loss of H2-K^d^ expression was not due to NHEJ-mediated deletions, but may reflect an epigenetic response to a DSB at an actively expressed gene [15]–[17]. This loss of expression was stable over several months of culture, and thus reflects an inadvertent but heritable genomic (or epigenomic) alteration that occurs as a side effect of TGC.

## Discussion

Targeted gene correction (TGC) has the potential to provide a powerful therapeutic approach for treatment of monogenic disorders. However, DSBs generated in the essential DNA cleavage step that targets gene correction can initiate mutations, translocations, and other forms of genomic instability. Here we show that DNA nicks can efficiently initiate TGC yet do not incur the deleterious repair events associated with DSBs.

One measure of the efficacy of gene correction is the ratio of TGC to mutagenesis at the target site. Nicks proved to initiate gene correction with 70-fold greater efficacy than DSBs at the target site. Moreover, nicks were associated with a considerably reduced frequency of off-target events. Notably, we found that DSB-initiated second-site events may not only reflect sequence loss but may also reflect epigenetic modification, as has previously been documented at DNA proximal to repaired DSBs [15]–[17]. The efficacy of nick-initiated TGC will thus be especially beneficial in a therapeutic context, where it may be difficult to monitor all off-target damage.

The efficiency of nick-initiated TGC was several-fold lower than that of DSB-initiated TGC, based on assays with two different reporters. Nicks are efficiently religated and this would be predicted to compete with homologous recombination to determine the frequency of TGC. Inhibition of religation may in the future boost frequencies of nick-initiated TGC without causing inadvertent off-target NHEJ and thus contribute to the efficacy of nick-initiated TGC.

### The mechanism of nick-initiated TGC

Several lines of evidence have suggested that nicks can initiate homologous recombination [18]–[20]. However, because a nick can be converted to a DSB upon DNA replication, it is difficult to exclude the possibility that recombination occurs not at the initiating nick, but at a resulting DSB. Our experiments with the Traffic Light reporter measured TGC (recombination) and NHEJ at a single I-AniI recognition site. If TGC depends upon conversion of a nick to a replicative DSB, then that DSB might be predicted to initiate not only TGC but also local mutagenesis, like a meganuclease-initiated DSB. However, we found that nicks initiated a much greater frequency of TGC than of NHEJ. This might reflect differences in repair mechanisms or pathways available to DSBs initiated by a meganuclease and DSBs resulting from DNA replication at a nick. Several factors may favor repair by homologous recombination of nicks that are converted to DSBs when encountered by a replication fork.. (1) Replicative DSBs are one-ended and may, therefore, be processed differently than two-ended DSBs like those created by a cleavase [eg.], [21, 22]. (2) DSBs in the context of a replication fork may be especially likely to take advantage of the homologous recombination machinery know to be involved in restart of stalled replication forks. (3) NHEJ activities are low in S phase [23], when a replicative DSB would undergo repair, whereas cleavase-induced DSBs may be repaired during the G1 phase of the cell cycle when NHEJ predominates.

**Figure 5:**
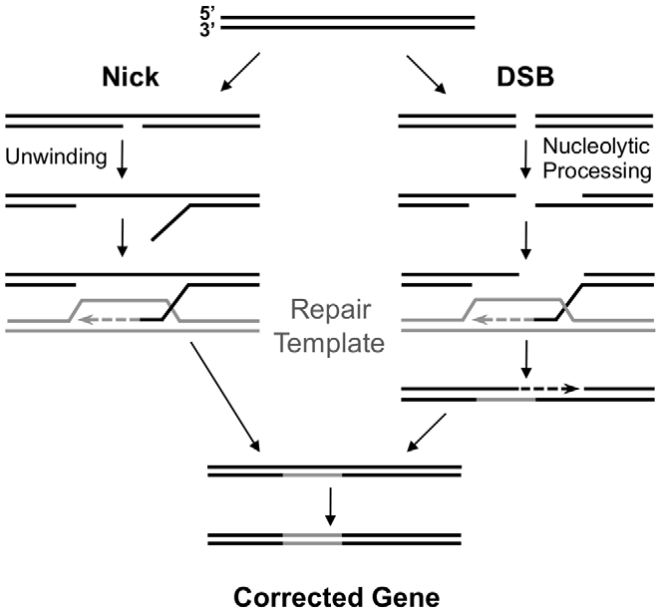
Model of TGC initiated by nicks or DSBs. A model of TGC initiated by a nick or a DSB. The processing of a nick to form an invading 3′ single-stranded end occurs by duplex unwinding (left); while a DSB undergoes nucleolytic processing (right). The next step involves invasion of the repair template (gray) and repair by a synthesis-dependent strand-annealing (SDSA) pathway. The resulting heteroduplex may then be corrected by the mismatch repair pathway or be resolved by replication and segregation, to generate a corrected gene.

Another possibility is that nicks may be able to initiate homologous recombination without proceeding through a DSB intermediate. In fact, many of the first models of homologous recombination postulated that DNA nicks could directly initiate recombination [see 24]. Figure 5 presents a model of TGC initiated directly at a nick or a DSB. One key difference between TGC initiated by nicks or DSBs is in the origin of the invading 3′ single-stranded end. Unwinding at a nick by a 3′-5′ helicase would yield a 3′ single-stranded end for strand invasion (Fig. 5, left). In contrast, nucleolytic processing is thought to yield these ends in DSB-initiated TGC (Fig. 5, right).

### Implications for therapy

The superior efficacy of nick-initiated TGC that we have documented has clear therapeutic implications, and should inform future design of all classes of meganucleases for targeted gene correction. Monomeric homing endonucleases contain distinct active sites that direct cleavage of the two DNA strands, and a cleavase can be readily converted to a nickase by inactivation of one site, as has been done not only for I-AniI [10] but also for I-SceI [25]. This conversion should also be readily achievable with engineered single-chain “homodimers” [26] and obligate heterodimeric homing endonucleases [27]. The recent design of nicking versions of FokI [28], combined with the rapid strategies for engineering custom zinc-finger nucleases [29] and TALE nucleases [30]–[32], should greatly expand the reach of nick-initiated TGC to the spectrum of enzymes currently being adapted to this purpose.

## Materials and Methods

### Plasmids and cell lines

The I-AniI expression constructs contained the previously described reoAni coding sequence [10] containing two mutations, F13Y and S111Y, that have been shown to enhance both DNA-binding and cleavage at physiological temperatures [33], incorporated into a vector bearing the EF1α promoter, T2A translational linker [13] and mTagBFP [12]. The I-AniI second site NHEJ reporter was created by modifying pCOH-CD4 [14] to replace the I-SceI site with an I-AniI site. Cloning details are provided as Supporting Information (Methods S1). All cell lines were derived from HEK293T, an SV40-transformed human embryonic kidney cell line. Lentiviral transductants bearing the Traffic Light reporter were kindly provided by Drs. Michael Certo and Andrew Scharenberg (Seattle Childrens Research Institute). Lines used in the two-reporter assays were created by lentiviral transduction with the TGC reporter followed by stable transfection with the second-site reporter and testing of individual clones for H2-K^d^ expression by flow cytometry. In all cases, results of the TGC assay were validated on multiple clones.

### Cell culture and transfection

The human embryonic kidney cell line 293T [34], [35] and its derivatives were grown at 37°C, 5% CO_2_ in Dulbecco-modified Eagle's medium (Hyclone) supplemented with 10% fetal bovine serum (Atlanta Biological, Lawrenceville, GA) and 200 units/ml penicillin, 200 µg/ml streptomycin (Hyclone) and 2 mM L-glutamine (Hyclone). Transfections were performed using Lipofectamine LTX (Invitrogen, Carlsbad, CA) according to the manufacturer's protocol. Briefly, cells were seeded in 2 ml (6-well plates) or 1 ml (12-well plates) of medium 24 hours prior to transfection at 2–2.5×10^5^ cells/ml and transfected with 1 µg of plasmid and 2.5 µl of Lipofectamine per ml. Transfection efficiency was typically measured by transfection with pEGFP-N1 control vector (Clontech, Mountain View, CA). For TGC, NHEJ and second-site reporter analysis, cells were expanded 1 day post-transfection and collected for analysis 3 days post-transfection.

### Flow cytometry

Viability was assayed 3 days post-transfection by staining with LIVE/DEAD® Fixable Dead Cell Stain; Invitrogen), which identifies dead cells based on loss of membrane integrity. For TGC, NHEJ and second-site reporter analysis, cells were fixed in 2% formaldehyde 3 days post-transfection and analyzed on a LSR II flow cytometer (Becton Dickinson, Franklin Lakes, NJ). H2-K^d^ and CD4 expression were detected by washing fixed cells twice with FACS buffer (PBS, 1% FBS, 0.1% soidum azide), resuspending in FACS buffer containing PE-conjugated anti-H2-K^d^ or anti-CD4 antibody (1∶500; (Becton Dickinson), incubating at 4°C in the dark for 1 hr, washing twice with FACS buffer and analyzing on a LSR II flow cytometer. Approximately 500,000 events were gated for linear side scatter and forward scatter to identify cells, and cells gated for linear forward scatter height and width to eliminate doublets. GFP, PE and mCherry fluorescence were detected with a 488 nm laser; mTagBFP fluorescence was detected with a 405 nm laser. Data was compensated and analyzed with FlowJo (Tree Star, Ashland, OR) flow cytometry analysis software.

### Data analysis

The data from each experiment used to calculate the means and SEMs for Fig. 1, 2, 3, and 4 are shown in Tables S1, S2, S3, S4. Statistical significance was determined by two-tailed t-test. Detailed descriptions of the calculations used to derive H2K^d^ loss frequencies are also described in the legend of Table S3.

## 

## Supporting Information

Figure S1 **NHEJ at the I-AniI site accounts for DSB-promoted mCherry^+^ cells.** Top line, flanking sequence (black) and I-AniI recognition motif (red) in the Traffic Light reporter. Below are sequences of 16 independent mutational events detected in a sorted mCherry^+^ population following transfection with an I-AniI cleavase expression construct and repair donor, identifying deletions (dashes) and heterologous or inserted nucleotides (blue). One event (+1) was a single bp insertion in the target site. Of the remaining events, eleven were simple deletions ranging in size from 2 bp to 353 bp; and four were more complex insertions/deletions removing a total of 8, 59, 218 or 395 bp. For example, one (Δ395) consisted of a 396 bp deletion with one bp insertion 39 bp downstream of the deletion junction. All these sequence alterations are consistent with production by the canonical NHEJ pathway. Because of the position of the I-AniI recognition sequence in the GFP gene, deletions of more than about 100 bp upstream or 600 bp downstream of the cut site would not be detected. Nonetheless, this does not seem to have significantly limited the types of events observed. Only 3 of the 15 deletions removed sequence upstream of the cut site (2, 6 and 33 bp); and while 6 of 15 deletions removed more than 100 bp of sequence downstream of the cleavage site, none removed more than 400 bp.
(TIF)

Figure S2 **Chromosomal TGC reporter.** This reporter was used in combination with the second-site reporter (Fig. 4A). It carries a PGK promoter (P-PGK) driving expression of a defective GFP gene carrying an I-AniI site and two stop codons. The repair template carries a defective GFP gene with an N-terminal deletion of 14 amino acids (Δ14) driven by the PGK promoter. Homologous recombination generates a corrected chromosomal GFP gene and renders cells GFP^+^.
(TIF)

Table S1 **Raw data used in** **Figure 1C** **(right).** Data from the total transfected population for each transfection of the Traffic Light reporter cell line performed with either donor only and no I-AniI expression or with catalytically inactive, nickase and cleavase I-AniI expression constructs and no donor are presented. Transfections done on the same day are indicated by suffix (*e.g.* 1A, 1B and 1C). The mean and standard error of the mean (SEM) are calculated for each class of transfections.
(DOC)

Table S2 **Raw data used in** **Figures 1C** **,** **2B, 2C** **and** **3** **.** Data for each transfection of the Traffic Light reporter cell line performed with catalytically inactive, nickase and cleavase I-AniI expression constructs and donor are presented. The first five column groupings are the data from each of the five expression quintiles used to generate Figures 2B, 2C and 3. The yellow highlighted cells are those in which the GFP∶mCherry ratio could not be calculated (denominator = 0) and were excluded from the calculation of mean and SEM for those quintiles. The last group of columns is the data from the total transfected population that was used to generate Figure 1C (left). Transfections done on the same day are indicated by suffix (*e.g.* 1A, 1B and 1C). The mean and standard error of the mean (SEM) are calculated for each class of transfections.
(PDF)

Table S3 **Raw data used in** **Figure 4B** **.** Data from the total transfected population for each set of transfections of the second-site reporter cell line EJ4GFP7 analyzed for H2K expression are presented. Each set consists of one transfection of catalytically inactive, nickase and cleavase I-AniI expression constructs plus donor. Below each set of transfections are the calculated frequency of H2K^d^ negative cells among GFP^+^ and GFP^−^; both raw (H2K^−^) and, for nickase and cleavase, with background subtracted (ΔH2K-). The percent of TGC (GFP^+^) and H2K^d^ loss for the nickase and cleavase is calculated below each set of H2K^−^ and ΔH2K^−^ values. H2K^d^ loss is calculated as ΔH2K- divided by the frequency of H2K+ cells and expressed as a percentage. The mean and standard error of the mean (SEM) of the H2K^d^ loss and TGC are calculated.
(DOC)

Table S4 **Raw data used in** **Figure 4C** **.** Data from the total transfected population for each set of transfections of the second-site reporter cell lines EJ2GFP15 and EJ4GFP7 analyzed for CD4 expression are presented. Each set consists of one transfection of catalytically inactive, nickase and cleavase I-AniI expression constructs plus donor. Below each set of transfections are the calculated frequencies (as percentage) of GFP^+^ cells as well as CD4^+^ among total cells and CD4^+^ among GFP^+^ cells. CD4^+^ frequencies with background subtracted are also calculated. The mean and standard error of the mean (SEM) of the frequencies of GFP^+^ cells and the background subtracted CD4^+^ frequencies are calculated.
(DOC)

Methods S1 **Cloning and plasmid construction details.**
(DOC)

## References

[1] Naldini L (2011). Ex vivo gene transfer and correction for cell-based therapies.. Nat Rev Genet.

[2] Pessach IM, Notarangelo LD (2011). Gene therapy for primary immunodeficiencies: Looking ahead, toward gene correction.. J Allergy Clin Immunol.

[3] Urnov FD, Miller JC, Lee YL, Beausejour CM, Rock JM (2005). Highly efficient endogenous human gene correction using designed zinc-finger nucleases.. Nature.

[4] Olsen PA, Gelazauskaite M, Randol M, Krauss S (2010). Analysis of illegitimate genomic integration mediated by zinc-finger nucleases: implications for specificity of targeted gene correction.. BMC Mol Biol.

[5] Petek LM, Russell DW, Miller DG (2010). Frequent endonuclease cleavage at off-target locations in vivo.. Mol Ther.

[6] Boch J (2011). TALEs of genome targeting.. Nat Biotechnol.

[7] Stoddard BL (2011). Homing endonucleases: from microbial genetic invaders to reagents for targeted DNA modification.. Structure.

[8] Urnov FD, Rebar EJ, Holmes MC, Zhang HS, Gregory PD (2010). Genome editing with engineered zinc finger nucleases.. Nat Rev Genet.

[9] Hacein-Bey-Abina S, von Kalle C, Schmidt M, Le Deist F, Wulffraat N (2003). A serious adverse event after successful gene therapy for X-linked severe combined immunodeficiency.. N Engl J Med.

[10] McConnell Smith A, Takeuchi R, Pellenz S, Davis L, Maizels N (2009). Generation of a nicking enzyme that stimulates site-specific gene conversion from the I-AniI LAGLIDADG homing endonuclease.. Proc Natl Acad Sci U S A.

[11] Certo MT, Ryu BY, Annis JE, Garibov M, Jarjour J (2011). Tracking genome engineering outcome at individual DNA breakpoints.. Nat Methods.

[12] Subach OM, Gundorov IS, Yoshimura M, Subach FV, Zhang J (2008). Conversion of red fluorescent protein into a bright blue probe.. Chem Biol.

[13] Szymczak AL, Workman CJ, Wang Y, Vignali KM, Dilioglou S (2004). Correction of multi-gene deficiency in vivo using a single ‘self-cleaving’ 2A peptide-based retroviral vector.. Nat Biotechnol.

[14] Guirouilh-Barbat J, Huck S, Bertrand P, Pirzio L, Desmaze C (2004). Impact of the KU80 pathway on NHEJ-induced genome rearrangements in mammalian cells.. Mol Cell.

[15] Cuozzo C, Porcellini A, Angrisano T, Morano A, Lee B (2007). DNA damage, homology-directed repair, and DNA methylation.. PLoS Genet.

[16] O'Hagan HM, Mohammad HP, Baylin SB (2008). Double strand breaks can initiate gene silencing and SIRT1-dependent onset of DNA methylation in an exogenous promoter CpG island.. PLoS Genet.

[17] Shanbhag NM, Rafalska-Metcalf IU, Balane-Bolivar C, Janicki SM, Greenberg RA (2010). ATM-dependent chromatin changes silence transcription in cis to DNA double-strand breaks.. Cell.

[18] Arcangioli B (1998). A site- and strand-specific DNA break confers asymmetric switching potential in fission yeast.. EMBO J.

[19] Lee GS, Neiditch MB, Salus SS, Roth DB (2004). RAG proteins shepherd double-strand breaks to a specific pathway, suppressing error-prone repair, but RAG nicking initiates homologous recombination.. Cell.

[20] Strathern JN, Weinstock KG, Higgins DR, McGill CB (1991). A novel recombinator in yeast based on gene II protein from bacteriophage f1.. Genetics.

[21] Allen C, Ashley AK, Hromas R, Nickoloff JA (2011). More forks on the road to replication stress recovery.. J Mol Cell Biol.

[22] Savolainen L, Helleday T (2009). Transcription-associated recombination is independent of XRCC2 and mechanistically separate from homology-directed DNA double-strand break repair.. Nucleic Acids Res.

[23] Rodrigue A, Lafrance M, Gauthier MC, McDonald D, Hendzel M (2006). Interplay between human DNA repair proteins at a unique double-strand break in vivo.. EMBO J.

[24] Smith GR (2004). How homologous recombination is initiated: unexpected evidence for single-strand nicks from V(D)J site-specific recombination.. Cell.

[25] Niu Y, Tenney K, Li H, Gimble FS (2008). Engineering variants of the I-SceI homing endonuclease with strand-specific and site-specific DNA-nicking activity.. J Mol Biol.

[26] Li H, Pellenz S, Ulge U, Stoddard BL, Monnat RJ (2009). Generation of single-chain LAGLIDADG homing endonucleases from native homodimeric precursor proteins.. Nucleic Acids Res.

[27] Fajardo-Sanchez E, Stricher F, Paques F, Isalan M, Serrano L (2008). Computer design of obligate heterodimer meganucleases allows efficient cutting of custom DNA sequences.. Nucleic Acids Res.

[28] Sanders KL, Catto LE, Bellamy SR, Halford SE (2009). Targeting individual subunits of the FokI restriction endonuclease to specific DNA strands.. Nucleic Acids Res.

[29] Maeder ML, Thibodeau-Beganny S, Osiak A, Wright DA, Anthony RM (2008). Rapid “open-source” engineering of customized zinc-finger nucleases for highly efficient gene modification.. Mol Cell.

[30] Christian M, Cermak T, Doyle EL, Schmidt C, Zhang F (2010). Targeting DNA double-strand breaks with TAL effector nucleases.. Genetics.

[31] Li T, Huang S, Jiang WZ, Wright D, Spalding MH (2010). TAL nucleases (TALNs): hybrid proteins composed of TAL effectors and FokI DNA-cleavage domain.. Nucleic Acids Res.

[32] Zhang F, Cong L, Lodato S, Kosuri S, Church GM (2011). Efficient construction of sequence-specific TAL effectors for modulating mammalian transcription.. Nat Biotechnol.

[33] Takeuchi R, Certo M, Caprara MG, Scharenberg AM, Stoddard BL (2009). Optimization of in vivo activity of a bifunctional homing endonuclease and maturase reverses evolutionary degradation.. Nucleic Acids Res.

[34] DuBridge RB, Tang P, Hsia HC, Leong PM, Miller JH (1987). Analysis of mutation in human cells by using an Epstein-Barr virus shuttle system.. Mol Cell Biol.

[35] Pear WS, Nolan GP, Scott ML, Baltimore D (1993). Production of high-titer helper-free retroviruses by transient transfection.. Proc Natl Acad Sci U S A.

