# Supplementary figures and images for: DNA Nicks Promote Efficient and Safe Targeted Gene Correction
Source: PLoS One. 2011 Sep 1;6(9):e23981. doi: 10.1371/journal.pone.0023981 (PMC3164693; doi:10.1371/journal.pone.0023981)

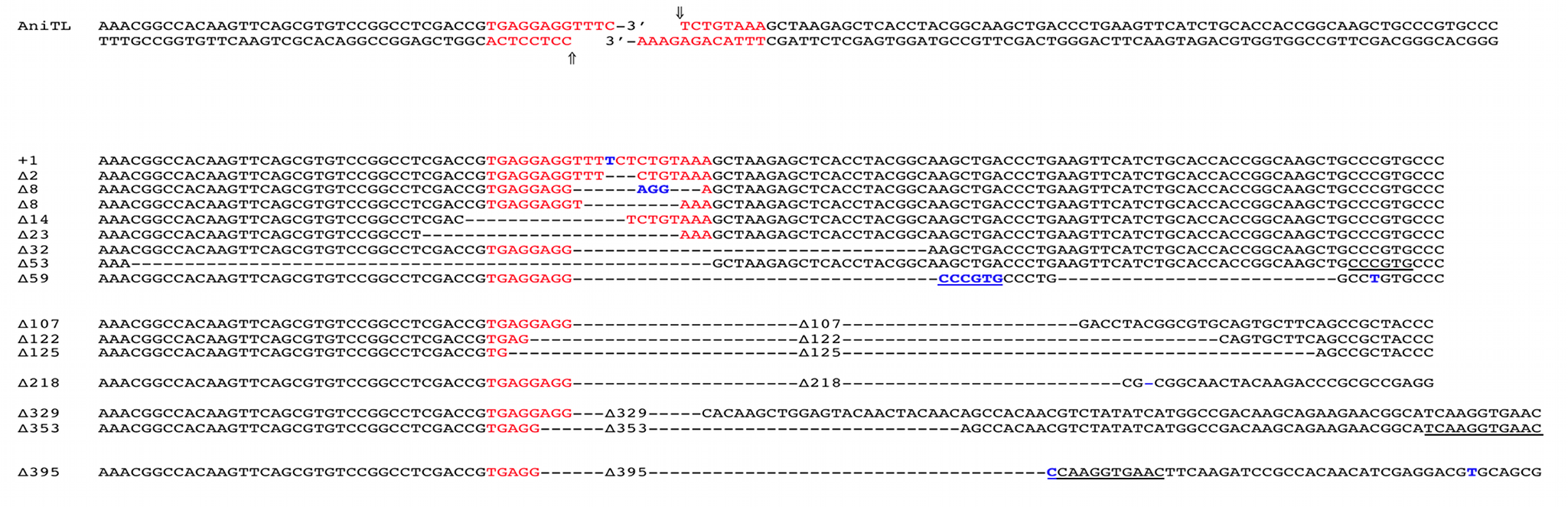

Supplement: Figure S1 — NHEJ at the I-AniI site accounts for DSB-promoted mCherry+ cells. Top line, flanking sequence (black) and I-AniI recognition motif (red) in the Traffic Light reporter. Below are sequences of 16 independent mutational events detected in a sorted mCherry+ population following transfection with an I-AniI cleavase expression construct and repair donor, identifying deletions (dashes) and heterologous or inserted nucleotides (blue). One event (+1) was a single bp insertion in the target site. Of the remaining events, eleven were simple deletions ranging in size from 2 bp to 353 bp; and four were more complex insertions/deletions removing a total of 8, 59, 218 or 395 bp. For example, one (Δ395) consisted of a 396 bp deletion with one bp insertion 39 bp downstream of the deletion junction. All these sequence alterations are consistent with production by the canonical NHEJ pathway. Because of the position of the I-AniI recognition sequence in the GFP gene, deletions of more than about 100 bp upstream or 600 bp downstream of the cut site would not be detected. Nonetheless, this does not seem to have significantly limited the types of events observed. Only 3 of the 15 deletions removed sequence upstream of the cut site (2, 6 and 33 bp); and while 6 of 15 deletions removed more than 100 bp of sequence downstream of the cleavage site, none removed more than 400 bp. (TIF) [file pone.0023981.s001.tif]

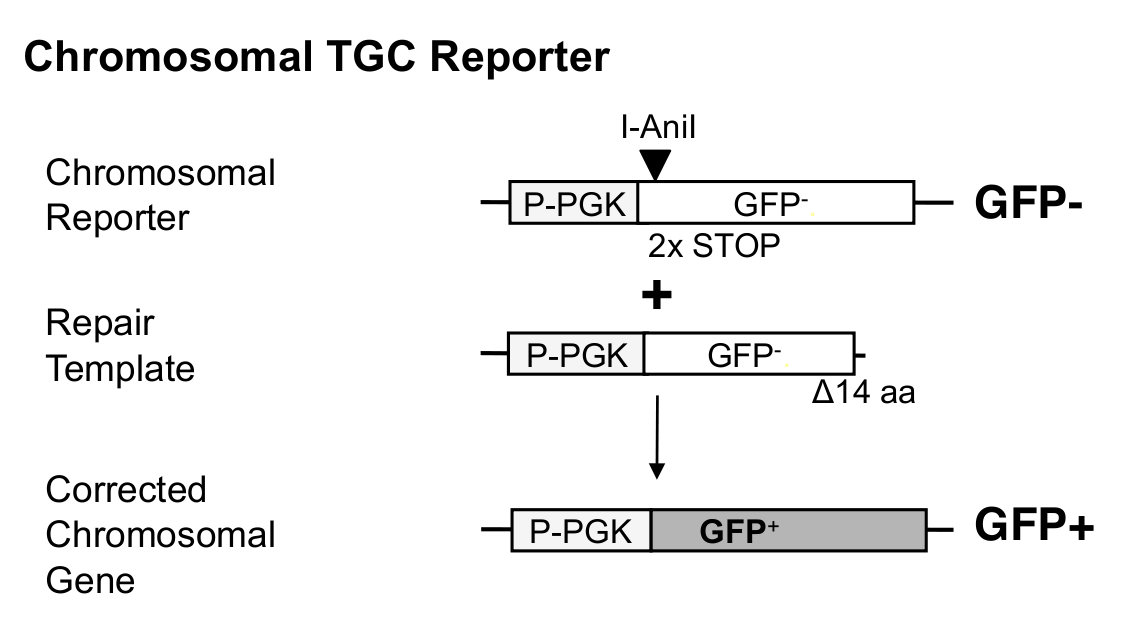

Supplement: Figure S2 — Chromosomal TGC reporter. This reporter was used in combination with the second-site reporter (Fig. 4A). It carries a PGK promoter (P-PGK) driving expression of a defective GFP gene carrying an I-AniI site and two stop codons. The repair template carries a defective GFP gene with an N-terminal deletion of 14 amino acids (Δ14) driven by the PGK promoter. Homologous recombination generates a corrected chromosomal GFP gene and renders cells GFP+. (TIF) [file pone.0023981.s002.tif]
