# Supplementary material for: DNA Nicks Promote Efficient and Safe Targeted Gene Correction
Source: PLoS One. 2011 Sep 1;6(9):e23981. doi: 10.1371/journal.pone.0023981 (PMC3164693; doi:10.1371/journal.pone.0023981)
Supplement: Table S1 — Raw data used in Figure 1C (right). Data from the total transfected population for each transfection of the Traffic Light reporter cell line performed with either donor only and no I-AniI expression or with catalytically inactive, nickase and cleavase I-AniI expression constructs and no donor are presented. Transfections done on the same day are indicated by suffix (e.g. 1A, 1B and 1C). The mean and standard error of the mean (SEM) are calculated for each class of transfections. (DOC) [file pone.0023981.s003.doc]

**Table S1.** **Raw data used in Figure 1C (right).**

| **Transfection** |  | **% GFP+ in total** | **% mCherry+ in total** |
| --- | --- | --- | --- |
|  |  |  |  |
| donor only-1 |  | 0 | 0 |
| donor only-2 |  | 7.14E-04 | 0 |
| donor only-3A |  | 7.24E-04 | 7.24E-04 |
| donor only-3B |  | 7.19E-04 | 0 |
|  |  |  |  |
|  | Mean | 0.001 | 0.000 |
|  | SEM | 0.000 | 0.000 |
|  |  |  |  |
|  |  |  |  |
| inactive_no donor-1A |  | 9.59E-04 | 0 |
| inactive_ no dono-1B |  | 8.07E-04 | 0 |
| inactive_no donor-1C |  | 0 | 4.42E-04 |
|  |  |  |  |
|  | Mean | 0.001 | 0.000 |
|  | SEM | 0.000 | 0.000 |
|  |  |  |  |
|  |  |  |  |
| nickase_no donor-1A |  | 8.31E-04 | 0.0166 |
| nickase_no donor-1B |  | 0 | 9.00E-03 |
| nickase_no donor-1C |  | 4.05E-04 | 8.51E-03 |
| nickase_no donor-2 |  | 0 | 4.14E-03 |
| nickase_no donor-3A |  | 7.39E-04 | 2.22E-03 |
| nickase_no donor-3B |  | 7.26E-04 | 2.91E-03 |
|  |  |  |  |
|  | Mean | 0.000 | 0.007 |
|  | SEM | 0.000 | 0.002 |
|  |  |  |  |
|  |  |  |  |
| cleavase_no donor-1A |  | 9.50E-04 | 0.989 |
| cleavase_no donor-1B |  | 0 | 0.981 |
| cleavase_no donor-1C |  | 4.45E-04 | 1.03 |
| cleavase_no donor-2 |  | 1.05E-03 | 0.513 |
| cleavase_no donor-3A |  | 3.90E-04 | 0.457 |
| cleavase_no donor-3B |  | 7.95E-04 | 0.43 |
|  |  |  |  |
|  | Mean | 0.001 | 0.733 |
|  | SEM | 0.000 | 0.120 |
