# Supplementary material for: DNA Nicks Promote Efficient and Safe Targeted Gene Correction
Source: PLoS One. 2011 Sep 1;6(9):e23981. doi: 10.1371/journal.pone.0023981 (PMC3164693; doi:10.1371/journal.pone.0023981)
Supplement: Table S2 — Raw data used in Figures 1C , 2B, 2C and 3 . Data for each transfection of the Traffic Light reporter cell line performed with catalytically inactive, nickase and cleavase I-AniI expression constructs and donor are presented. The first five column groupings are the data from each of the five expression quintiles used to generate Figures 2B, 2C and 3. The yellow highlighted cells are those in which the GFP∶mCherry ratio could not be calculated (denominator = 0) and were excluded from the calculation of mean and SEM for those quintiles. The last group of columns is the data from the total transfected population that was used to generate Figure 1C (left). Transfections done on the same day are indicated by suffix (e.g. 1A, 1B and 1C). The mean and standard error of the mean (SEM) are calculated for each class of transfections. (PDF) [file pone.0023981.s004.pdf]

Table S2. Raw data used in Figures 1C, 2B, 2C and 3.

	quintile 1			quintile 2			quintile 3			quintile 4			quintile 5			total population		
Transfection	% GFP+ in Quintile 1	% mCherry+ in Quintile 1	GFP:mCherry ratio	% GFP+ in Quintile 2	% mCherry+ in Quintile 2	GFP:mCherry ratio	% GFP+ in Quintile 3	% mCherry+ in Quintile 3	GFP:mCherry ratio	% GFP+ in Quintile 4	% mCherry+ in Quintile 4	GFP:mCherry ratio	% GFP+ in Quintile 5	% mCherry+ in Quintile 5	GFP:mCherry ratio	% GFP+ in total	% mCherry+ in total	GFP:mCherry ratio
inactive + donor-1A	0	0		0	6.90E-03		0	0		0	0		0	7.28E-03		0	1.52E-03	
inactive + donor-1B	0	0		0	0		0	0		0	0		7.73E-03	0		8.20E-04	0	
inactive + donor-1C	0	0		0	0		0	0		5.48E-03	0		3.25E-03	0		1.47E-03	0	
inactive + donor-2A	0	0		0	2.42E-03		0	0		0	0		7.88E-03	7.88E-03		6.88E-04	1.38E-03	
inactive + donor-2B	0	0		0	2.79E-03		0	0.0169		0	0		0.0159	0		1.35E-03	1.35E-03	
inactive + donor-3A	0	0		0	0		0	0		0	0		8.35E-03	0		7.43E-04	0	
inactive + donor-3B	0	0		0	0		0	0		3.15E-03	0		4.42E-03	0		7.65E-04	0	
inactive + donor-3C	0	0		0	0		0	0		0	0		0	0		0	0	
Mean	0.000	0.000		0.000	0.002		0.000	0.002		0.001	0.000		0.006	0.002		0.001	0.001	
SEM	0.000	0.000		0.000	0.001		0.000	0.002		0.001	0.000		0.002	0.001		0.000	0.000	
nickase + donor-1A	0	0	#DIV/0!	0	0	#DIV/0!	0	0.0128	0.000	0.122	0.0136	8.971	0.347	0	#DIV/0!	0.0707	4.21E-03	16.793
nickase + donor-1B	0	0	#DIV/0!	0	0	#DIV/0!	0.0525	0.0131	4.008	0.114	0.0131	8.702	0.524	0.0204	25.686	0.0896	5.81E-03	15.422
nickase + donor-1C	0	5.48E-03	0.000	0	2.56E-03	0.000	6.27E-03	0.0188	0.334	0.114	0.0193	5.907	0.404	0.016	25.250	0.073	9.42E-03	7.749
nickase + donor-2A	0	0	#DIV/0!	0	0	#DIV/0!	7.31E-03	0	#DIV/0!	0.0748	9.35E-03	8.000	0.315	0.0109	28.899	0.0412	2.10E-03	19.619
nickase + donor-2B	0	2.13E-03	0.000	0	9.99E-04	0.000	0	0	#DIV/0!	0.114	0.0252	4.524	0.3	7.42E-03	40.431	0.0428	4.52E-03	9.469
nickase + donor-3A	0	0	#DIV/0!	1.55E-03	0	#DIV/0!	9.43E-03	0.0283	0.333	0.0611	3.22E-03	18.975	0.243	4.26E-03	57.042	0.0305	2.20E-03	13.864
nickase + donor-3B	0	0	#DIV/0!	0	0	#DIV/0!	0.0156	7.79E-03	2.003	0.0532	0.0125	4.256	0.217	0.0123	17.642	0.0272	3.35E-03	8.119
nickase + donor-3C	0	4.18E-03	0.000	0	2.31E-03	0.000	7.80E-03	0	#DIV/0!	0.066	0.0126	5.238	0.227	0.029	7.828	0.0293	6.31E-03	4.643
Mean	0.000	0.001	#DIV/0!	0.000	0.001	#DIV/0!	0.012	0.010	1.335	0.090	0.014	8.072	0.322	0.013	28.968	0.051	0.005	11.960
SEM	0.000	0.001	#DIV/0!	0.000	0.000	#DIV/0!	0.006	0.004	0.754	0.010	0.002	1.690	0.037	0.003	6.015	0.008	0.001	1.841
cleavase + donor-1A	9.99E-03	0.145	0.069	0.128	1.31	0.098	0.771	6.21	0.124	1.27	7.53	0.169	1.45	4.2	0.345	0.198	1.15	0.172
cleavase + donor-1B	8.72E-03	0.166	0.053	0.147	1.61	0.091	1.04	6.42	0.162	1.13	7.26	0.156	1.33	3.81	0.349	0.19	1.17	0.162
cleavase + donor-1C	9.19E-03	0.095	0.097	0.104	1.17	0.089	0.762	5.23	0.146	1.45	6.27	0.231	1.27	3.44	0.369	0.175	0.877	0.200
cleavase + donor-2A	4.42E-03	0.0379	0.117	0.0578	0.32	0.181	1.05	4.52	0.232	1.84	6.71	0.274	1.4	3.5	0.400	0.153	0.587	0.261
cleavase + donor-2B	2.84E-03	0.0418	0.068	0.0484	0.322	0.150	0.84	4.39	0.191	1.62	6.21	0.261	1.17	3.45	0.339	0.145	0.627	0.231
cleavase + donor-3A	2.60E-03	0.0301	0.086	0.0841	0.41	0.205	1.08	3.4	0.318	1.49	4.48	0.333	1.35	2.28	0.592	0.139	0.411	0.338
cleavase + donor-3B	6.32E-04	0.0316	0.020	0.0713	0.419	0.170	0.824	3.74	0.220	1.46	4.97	0.294	0.998	2.21	0.452	0.121	0.476	0.254
cleavase + donor-3C	1.83E-03	0.0286	0.064	0.0472	0.318	0.148	0.829	3.15	0.263	1.31	4.37	0.300	0.976	2.04	0.478	0.112	0.39	0.287
Mean	0.005	0.072	0.072	0.086	0.735	0.142	0.900	4.633	0.207	1.446	5.975	0.252	1.243	3.116	0.416	0.154	0.711	0.238
SEM	0.001	0.020	0.010	0.013	0.189	0.016	0.047	0.436	0.023	0.078	0.435	0.022	0.063	0.290	0.031	0.011	0.112	0.021
