# Supplementary material for: DNA Nicks Promote Efficient and Safe Targeted Gene Correction
Source: PLoS One. 2011 Sep 1;6(9):e23981. doi: 10.1371/journal.pone.0023981 (PMC3164693; doi:10.1371/journal.pone.0023981)
Supplement: Table S3 — Raw data used in Figure 4B . Data from the total transfected population for each set of transfections of the second-site reporter cell line EJ4GFP7 analyzed for H2K expression are presented. Each set consists of one transfection of catalytically inactive, nickase and cleavase I-AniI expression constructs plus donor. Below each set of transfections are the calculated frequency of H2Kd negative cells among GFP+ and GFP−; both raw (H2K−) and, for nickase and cleavase, with background subtracted (ΔH2K-). The percent of TGC (GFP+) and H2Kd loss for the nickase and cleavase is calculated below each set of H2K− and ΔH2K− values. H2Kd loss is calculated as ΔH2K- divided by the frequency of H2K+ cells and expressed as a percentage. The mean and standard error of the mean (SEM) of the H2Kd loss and TGC are calculated. (DOC) [file pone.0023981.s005.doc]

**Table S3. Raw data used in Figure 4B.**

| **experiment** |  |  |  |  |  |
| --- | --- | --- | --- | --- | --- |
|  |  | **%GFP+ H2K-** | **%GFP+ H2K+** | **%GFP- H2K+** | **%GFP- H2K-** |
| inactive A |  | 8.57E-03 | 0.0143 | 92.8 | 7.17 |
| nickase A |  | 0.0236 | 0.0843 | 91.4 | 8.53 |
| cleavase A |  | 0.365 | 0.0407 | 74.9 | 24.7 |
|  |  |  |  |  |  |
|  |  | GFP+ | GFP+ | GFP- | GFP- |
|  |  | ∆H2K- | H2K- | ∆H2K- | H2K- |
|  | inactive |  | 0.375 |  | 0.072 |
|  | nickase | 0.147 | 0.219 | 0.014 | 0.085 |
|  | cleavase | 0.828 | 0.900 | 0.176 | 0.248 |
|  |  |  |  |  |  |
|  |  |  | H2K | loss |  |
|  |  |  | among GFP+ | among GFP- | TGC+ |
|  |  |  |  |  | 0.02 |
|  |  | nickase | 15.8 | 1.5 | 0.11 |
|  |  | cleavase | 89.2 | 19.0 | 0.41 |
|  |  |  |  |  |  |
|  |  | **%GFP+ H2K-** | **%GFP+ H2K+** | **%GFP- H2K+** | **%GFP- H2K-** |
| inactive B |  | 2.77E-03 | 1.84E-03 | 92.8 | 7.17 |
| nickase B |  | 0.0112 | 0.079 | 91.8 | 8.14 |
| cleavase B |  | 0.709 | 0.0823 | 74.2 | 25 |
|  |  |  |  |  |  |
|  |  | GFP+ | GFP+ | GFP- | GFP- |
|  |  | ∆H2K- | H2K- | ∆H2K- | H2K- |
|  | inactive |  | 0.601 |  | 0.072 |
|  | nickase | 0.052 | 0.124 | 0.010 | 0.081 |
|  | cleavase | 0.824 | 0.896 | 0.180 | 0.252 |
|  |  |  |  |  |  |
|  |  |  | H2K | loss |  |
|  |  |  | among GFP+ | among GFP- | TGC+ |
|  |  |  |  |  | 0.00 |
|  |  | nickase | 5.7 | 1.0 | 0.09 |
|  |  | cleavase | 88.8 | 19.4 | 0.79 |
|  |  |  |  |  |  |
|  |  | **%GFP+ H2K-** | **%GFP+ H2K+** | **%GFP- H2K+** | **%GFP- H2K-** |
| inactive C |  | 8.09E-04 | 0.0231 | 92.5 | 7.48 |
| nickase C |  | 0.0259 | 0.13 | 90.8 | 9.06 |
| cleavase C |  | 0.549 | 0.0743 | 68.2 | 31.2 |
|  |  |  |  |  |  |
|  |  | GFP+ | GFP+ | GFP- | GFP- |
|  |  | ∆H2K- | H2K- | ∆H2K- | H2K- |
|  | inactive |  | 0.034 |  | 0.075 |
|  | nickase | 0.091 | 0.166 | 0.016 | 0.091 |
|  | cleavase | 0.806 | 0.881 | 0.239 | 0.314 |
|  |  |  |  |  |  |
|  |  |  | H2K | loss |  |
|  |  |  | among GFP+ | among GFP- | TGC+ |
|  |  |  |  |  | 0.02 |
|  |  | nickase | 9.9 | 1.7 | 0.16 |
|  |  | cleavase | 87.1 | 25.8 | 0.62 |
|  |  |  |  |  |  |
|  |  |  |  |  |  |
|  |  | **%GFP+ H2K-** | **%GFP+ H2K+** | **%GFP- H2K+** | **%GFP- H2K-** |
| inactive D |  | 1.52E-03 | 0.0164 | 92.4 | 7.54 |
| nickase D |  | 0.0215 | 0.123 | 91.4 | 8.43 |
| cleavase D |  | 1.12 | 0.159 | 68.2 | 30.5 |
|  |  |  |  |  |  |
|  |  | GFP+ | GFP+ | GFP- | GFP- |
|  |  | ∆H2K- | H2K- | ∆H2K- | H2K- |
|  | inactive |  | 0.085 |  | 0.075 |
|  | nickase | 0.073 | 0.149 | 0.009 | 0.084 |
|  | cleavase | 0.800 | 0.876 | 0.234 | 0.309 |
|  |  |  |  |  |  |
|  |  |  | H2K | loss |  |
|  |  |  | among GFP+ | among GFP- | TGC+ |
|  |  |  |  |  | 0.02 |
|  |  | nickase | 7.9 | 1.0 | 0.14 |
|  |  | cleavase | 86.6 | 25.3 | 1.28 |
|  |  |  |  |  |  |
|  |  | **%GFP+ H2K-** | **%GFP+ H2K+** | **%GFP- H2K+** | **%GFP- H2K-** |
| inactive E |  | 3.20E-03 | 3.78E-03 | 92.3 | 7.67 |
| nickase E |  | 0.0172 | 0.0702 | 92 | 7.87 |
| cleavase E |  | 0.9 | 0.127 | 76.1 | 22.9 |
|  |  |  |  |  |  |
|  |  | GFP+ | GFP+ | GFP- | GFP- |
|  |  | ∆H2K- | H2K- | ∆H2K- | H2K- |
|  | inactive |  | 0.458 |  | 0.077 |
|  | nickase | 0.120 | 0.197 | 0.002 | 0.079 |
|  | cleavase | 0.800 | 0.876 | 0.155 | 0.231 |
|  |  |  |  |  |  |
|  |  |  | H2K | loss |  |
|  |  |  | among GFP+ | among GFP- | TGC+ |
|  |  |  |  |  | 0.01 |
|  |  | nickase | 13.0 | 0.2 | 0.09 |
|  |  | cleavase | 86.6 | 16.7 | 1.03 |
|  |  |  |  |  |  |
|  |  | **%GFP+ H2K-** | **%GFP+ H2K+** | **%GFP- H2K+** | **%GFP- H2K-** |
| inactive F |  | 5.70E-04 | 2.28E-03 | 92.8 | 7.21 |
| nickase F |  | 0.0318 | 0.108 | 91.9 | 8 |
| cleavase F |  | 0.761 | 0.0955 | 75.2 | 24 |
|  |  |  |  |  |  |
|  |  | GFP+ | GFP+ | GFP- | GFP- |
|  |  | ∆H2K- | H2K- | ∆H2K- | H2K- |
|  | inactive |  | 0.200 |  | 0.072 |
|  | nickase | 0.155 | 0.227 | 0.008 | 0.080 |
|  | cleavase | 0.816 | 0.888 | 0.170 | 0.242 |
|  |  |  |  |  |  |
|  |  |  | H2K | loss |  |
|  |  |  | among GFP+ | among GFP- | TGC+ |
|  |  |  |  |  | 0.00 |
|  |  | nickase | 16.7 | 0.9 | 0.14 |
|  |  | cleavase | 88.0 | 18.3 | 0.86 |
|  |  |  |  |  |  |
|  |  |  |  |  |  |
|  |  |  | H2K | loss |  |
| I-AniI |  |  | among GFP+ | among GFP- | TGC+ |
| nickase |  | **mean** | 11.509 | 1.050 | 0.121 |
|  |  | **SEM** | 1.809 | 0.212 | 0.012 |
|  |  |  |  |  |  |
| cleavase |  | **mean** | 87.731 | 20.766 | 0.830 |
|  |  | **SEM** | 0.458 | 1.563 | 0.125 |
