# Supplementary material for: DNA Nicks Promote Efficient and Safe Targeted Gene Correction
Source: PLoS One. 2011 Sep 1;6(9):e23981. doi: 10.1371/journal.pone.0023981 (PMC3164693; doi:10.1371/journal.pone.0023981)
Supplement: Table S4 — Raw data used in Figure 4C . Data from the total transfected population for each set of transfections of the second-site reporter cell lines EJ2GFP15 and EJ4GFP7 analyzed for CD4 expression are presented. Each set consists of one transfection of catalytically inactive, nickase and cleavase I-AniI expression constructs plus donor. Below each set of transfections are the calculated frequencies (as percentage) of GFP+ cells as well as CD4+ among total cells and CD4+ among GFP+ cells. CD4+ frequencies with background subtracted are also calculated. The mean and standard error of the mean (SEM) of the frequencies of GFP+ cells and the background subtracted CD4+ frequencies are calculated. (DOC) [file pone.0023981.s006.doc]

**Table S4. Raw data used in Figure 4C.**

| **Experiment** |  |  |  |  |  |
| --- | --- | --- | --- | --- | --- |
|  |  | **%GFP+ CD4-** | **%GFP+ CD4+** | **%GFP- CD4+** | **%GFP- CD4-** |
| 2-15_A_nickase |  | 0.0972 | 1.59E-03 | 0.503 | 99.4 |
| 2-15_A_inactive |  | 2.56E-03 | 0.00E+00 | 0.527 | 99.5 |
| 2-15_A_cleavase |  | 2.04 | 0.0887 | 0.852 | 97 |
|  |  |  |  |  |  |
|  |  |  | **cleavase** | **nickase** | **inactive** |
| % GFP+ |  |  | 2.129 | 0.099 | 0.003 |
|  |  |  |  |  |  |
| %CD4+ (of total) |  |  | 0.941 | 0.505 | 0.527 |
| adjusted for dead |  |  | 0.414 | -0.022 |  |
|  |  |  |  |  |  |
| %CD4+ (of GFP+) |  |  | 4.167 | 1.609 | 0.000 |
| adjusted for dead |  |  | 3.640 | 1.083 |  |
|  |  |  |  |  |  |
|  |  | **%GFP+ CD4-** | **%GFP+ CD4+** | **%GFP- CD4+** | **%GFP- CD4-** |
| 2-15_B_nickase |  | 0.167 | 1.11E-03 | 0.564 | 99.3 |
| 2-15_B_inactive |  | 2.95E-03 | 8.05E-04 | 0.627 | 99.4 |
| 2-15_B_cleavase |  | 1.94 | 0.0811 | 1.02 | 97 |
|  |  |  |  |  |  |
|  |  |  | **cleavase** | **nickase** | **inactive** |
| % GFP+ |  |  | 2.020 | 0.168 | 0.004 |
|  |  |  |  |  |  |
| %CD4+ (of total) |  |  | 1.101 | 0.565 | 0.628 |
| adjusted for dead |  |  | 0.473 | -0.063 |  |
|  |  |  |  |  |  |
| %CD4+ (of GFP+) |  |  | 4.013 | 0.660 | 21.438 |
| adjusted for dead |  |  | 3.385 | 0.033 |  |
|  |  |  |  |  |  |
|  |  | **%GFP+ CD4-** | **%GFP+ CD4+** | **%GFP- CD4+** | **%GFP- CD4-** |
| 4-7_A_nickase |  | 0.0832 | 3.46E-04 | 0.064 | 99.9 |
| 4-7_A_inactive |  | 3.19E-03 | 2.90E-04 | 0.0542 | 99.9 |
| 4-7_A_cleavase |  | 1.01 | 0.0213 | 0.456 | 98.5 |
|  |  |  |  |  |  |
|  |  |  | **cleavase** | **nickase** | **inactive** |
| % GFP+ |  |  | 1.031 | 0.084 | 0.003 |
|  |  |  |  |  |  |
| %CD4+ (of total) |  |  | 0.477 | 0.064 | 0.055 |
| adjusted for dead |  |  | 0.423 | 0.010 |  |
|  |  |  |  |  |  |
| %CD4+ (of GFP+) |  |  | 2.065 | 0.414 | 8.333 |
| adjusted for dead |  |  | 2.011 | 0.360 |  |
|  |  |  |  |  |  |
|  |  | **%GFP+ CD4-** | **%GFP+ CD4+** | **%GFP- CD4+** | **%GFP- CD4-** |
| 4-7_B_nickase |  | 0.135 | 1.81E-04 | 0.112 | 99.8 |
| 4-7_B_inactive |  | 1.70E-03 | 0.00E+00 | 0.067 | 99.9 |
| 4-7_B_cleavase |  | 0.816 | 0.0264 | 0.623 | 98.5 |
|  |  |  |  |  |  |
|  |  |  | **cleavase** | **nickase** | **inactive** |
| % GFP+ |  |  | 0.843 | 0.135 | 0.002 |
|  |  |  |  |  |  |
| %CD4+ (of total) |  |  | 0.650 | 0.112 | 0.067 |
| adjusted for dead |  |  | 0.583 | 0.045 |  |
|  |  |  |  |  |  |
| %CD4+ (of GFP+) |  |  | 3.134 | 0.134 | 0.000 |
| adjusted for dead |  |  | 3.067 | 0.067 |  |
|  |  |  |  |  |  |
|  |  |  |  |  |  |
|  | **Mean** |  |  | **I-AniI** |  |
|  |  |  | **cleavase** | **nickase** | **inactive** |
|  | %GFP | mean | 1.506 | 0.121 | 0.003 |
|  |  | SEM | 0.331 | 0.019 | 0.000 |
|  |  |  |  |  |  |
|  | %CD4+ |  |  |  |  |
|  | (of total) | mean | 0.473 | -0.008 | #DIV/0! |
|  |  | SEM | 0.039 | 0.023 | #DIV/0! |
|  |  |  |  |  |  |
|  | %CD4+ |  |  |  |  |
|  | (of GFP+) | mean | 3.026 | 0.385 | #DIV/0! |
|  |  | SEM | 0.358 | 0.244 | #DIV/0! |
