# Supplementary material for: DNA Nicks Promote Efficient and Safe Targeted Gene Correction
Source: PLoS One. 2011 Sep 1;6(9):e23981. doi: 10.1371/journal.pone.0023981 (PMC3164693; doi:10.1371/journal.pone.0023981)
Supplement: Methods S1 — Cloning and plasmid construction details. (DOC) [file pone.0023981.s007.doc]

# Methods S1

**Plasmids.** I-AniI expression constructs for use with the Traffic Light reporter were constructed by removing the donor GFP present in the three I-AniI expression constructs described below. The donor GFP was removed by digesting the plasmids with PacI and SalI, treating with the Klenow fragment of E. coli DNA polymerase I to create blunt ends, and religating.

The integrated I-AniI-initiated TGC reporter and I-AniI coding plasmid and GFP donor plasmid were modifications of pRSCSMPG'IScew2 and pRSCSIP∆14Gw2 (provided by Drs. Nina Munoz and Hans-Peter Kiem Fred Hutchinson Cancer Research Center, Seattle, WA). The I-SceI cut site of pRSCSMPG'IScew2 was digested with I-SceI (New England BioLabs) and the annealed oligonucleotides AniCut.F1:AniCut.R1 were ligated into the I-SceI site creating an I-AniI cut site (and recreating the I-SceI site). The resulting plasmid, pRSCSMPG'ISce_AniCS_w2, was used to make self-inactivating lentivirus. Lentivirus was provided by Dr. Byoung Ryu (Seattle Children’s Hospital, Seattle, WA).

I-AniI expression plasmids were made by modifying pRSCSIP∆14Gw2 as follows. The I-AniI-2A-mTagBFP was placed downstream of the Ef1α promoter as follows: the Ef1α promoter was amplified by PCR using primers NM1 and NM2. Both the cleavase and nickase versions of the I-AniI-2A-mTagBFP fusion ORFs were amplified by PCR using primers SbfI_reoY2-F and PacI_mTagBFP-R. Plasmids pCVL.SFFV.reoY2.2A.TagBFP.O-PRE and pCVL.SFFV.reoY2K227M.2A.TagBFP.O-PRE were used as template and provided by Dr. Byoung Ryu (Seattle Children’s Hospital, Seattle, WA). The pEf1α promoter PCR product was digested with SalI and SbfI and used to replace the XhoI – SbfI fragment of pRSCSIP∆14Gw2 containing the SFFV promoter. The I-AniI-2A-mTagBFP PCR products were digested with SbfI and PacI and used to replace the SbfI – PacI fragment containing I-SceI creating pRSCSIP_EF1a_AniY22AmTagBFP∆14Gw2 (cleavase) and pRSCSIP_EF1a_AniY2K227M2AmTagBFP∆14Gw2 (nickase). Catalytically inactive I-AniI, was constructed by QuickChange of the nickase plasmid using oligonucleotide AniQC(Q171K), and its complement, and contains an additional Q171K mutation.

The GFP donor plasmid used in Fig. 4 was made by inactivating the I-SceI gene in pRSCSIP∆14Gw2; the unique ClaI restriction site within the I-SceI coding sequence was digested, filled in with Klenow and religated creating a frameshift mutation.

The I-AniI-initiated NHEJ reporter was created by modifying pCOH-CD4 (13). The I-SceI cut sites of pCOH were partially digested with I-SceI (New England BioLabs) and a full-length (singly cut) plasmid isolated by gel-purification. The annealed oligonucleotides AniWT-F1:AniWT-R1 were ligated into the I-SceI site creating an I-AniI cut site (and destroying the I-SceI site). The remaining I-SceI site was digested and the annealed oligonucleotides AniWT-F1:AniWT-R1 were ligated into the I-SceI site creating plasmid pCOH-2xAni#7. Sequencing of pCOH-2xAni#7 revealed that two tandem I-AniI sites were ligated into the I-SceI proximal to the CD4 ORF while only a single I-AniI site is present between the CMV promoter and H2K^d^ ORF.

**Oligonucleotides:**

AniCut.F1: 5’- GGT GAG GAG GTT ACT CTG TTA TAG GGA TAA - 3’

AniCut.R1: 5’- CCC TAT AAC AGA GTA ACC TCC TCA CCT TAT - 3’

NM1: 5’- CCC GTC GAC CGT GAG GCT CCG GTG CCC - 3’

NM2: 5’- CCC CTC GAG TAG TTA TTA ATA GTA ATC AAT TAC G - 3’

SbfI_reoY2-F: 5’ – caa cct gca ggc cac cAT GGG ATA TCC ATA C - 3’

PacI_mTagBFP-R: 5’ – cca ctt aat taa ttc gaa TCA ATT AAG CTT GTG CCC CAG - 3’

AniQC(Q171K): 5’-GAT AGC TAG CTT TGA CAT TGC AAA AAG AGA TGG GGA TAT TTT AAT ATC AGC G-3’

AniWT-F1: 5’-TTA CAG AGA AAC CTC CTC ATT AT-3’

AniWT-R1: 5’-TGA GGA GGT TTC TCT GTA AAT AA-3’
